# Can seawater desalination be a win-win fix to our water cycle?

**DOI:** 10.1016/j.watres.2020.115906

**Published:** 2020-09-01

**Authors:** A. Pistocchi, T. Bleninger, C. Breyer, U. Caldera, C. Dorati, D. Ganora, M.M. Millán, C. Paton, D. Poullis, F. Salas Herrero, M. Sapiano, R. Semiat, C. Sommariva, S. Yuece, G. Zaragoza

**Affiliations:** a European Commission, Joint Research Centre, Italy; b Federal University of Parana’, Brazil; c LUT University, Finland; d Politecnico di Torino, Italy; e CEAM, Spain; f Seawater Greenhouse, UK; g Water Development Department, Cyprus; h Water and Energy Agency, Malta; i Technion, Israel; j SWPC, United Arab Emirates; k RWTH Aachen, Germany; l CIEMAT, Spain

**Keywords:** Desalination, Renewable energy, Water reuse, Precipitation recycling, Brine disposal, Resource recovery, Cost recovery

## Abstract

While we increasingly turn to desalination as a secure water supply, it is still perceived as an expensive and environmentally damaging solution, affordable only for affluent societies. In this contribution, we recast desalination from one of a last resort to a far-reaching, climate change mitigating, water security solution.

First, we argue that the benefits of desalination go beyond the single-use value of the water produced. If coupled with water reuse for irrigation, desalination reduces groundwater abstraction and augments the water cycle. As such, it may support both adaptation to, and mitigation of climate change impacts by deploying plentiful water for human use, with all the benefits that entails, while helping preserve and restore ecosystems.

Second, we counter two arguments commonly raised against desalination, namely its environmental impact and high cost.

The environmental impact can be fully controlled so as not to pose long-term threats, if driven by renewable energy. Desalination may then have a zero carbon footprint. Moreover, appropriately designed outfalls make the disposal of brine at sea compatible with marine ecosystems.. Recovery of energy, minerals and more water from brine reject (particularly in the form of vapour for cooling to enable more crops and vegetation to grow), while possible, is often hardly economically justified. However, resource recovery may become more attractive in the future, and help reduce the brine volumes to dispose of.

When fresh water becomes scarce, its cost tends to go up, making desalination increasingly economic. Moreover, desalination can have virtually no environmental costs. Considering the environmental costs of over-abstraction of freshwater, desalination tilts the balance in its favour.

## Highlights

• Here we recast desalination as a “win-win solution” to restore the water cycle.
• Desalination to become mainstream as societies understand its broader benefits.
• Coupling with water reuse maximizes socioeconomic/ecological return on investments.

## 1 Introduction

Desalination is usually regarded as an extreme solution for water supply, to be implemented in the absence of alternatives. The political discussion is usually focused on its affordability, while the scientific literature usually addresses it as a technological problem, requiring minimization of costs, energy use and impacts typically with a focus on the individual plant. This contribution aims at a more general discussion of the role of desalination in the future of water resources. While desalination and water reuse are both advocated as independent solutions to water scarcity, the importance of having them work together has not been sufficiently highlighted, and the role of desalination in truly replenishing the terrestrial water cycle has not been sufficiently noted.

In the policy debate, desalination is often seen as a threat for its high energy requirements, but it has not been sufficiently noted that it can be 100% decarbonized as highlighted in recent systematic assessments.

Brine disposal is often rightly described as a potential source of impact on marine ecosystems. Yet it should be clearly stressed that the impacts are well manageable. On the other hand, some overestimate the economic value of the brine’s mineral and energy content. While extraction of these resources can be feasible in some cases, it is unlikely to offset the cost of desalination. Nevertheless, it may be of significant help in the disposal of brine in certain circumstances.

Rather than focus on specialized features of desalination processes or technologies, the following sections critically discuss these aspects before establishing connections between the various parts of the problem in the conclusions.

## 2 Desalination supplements the water cycle beyond direct water supply

**Fig. 1:**
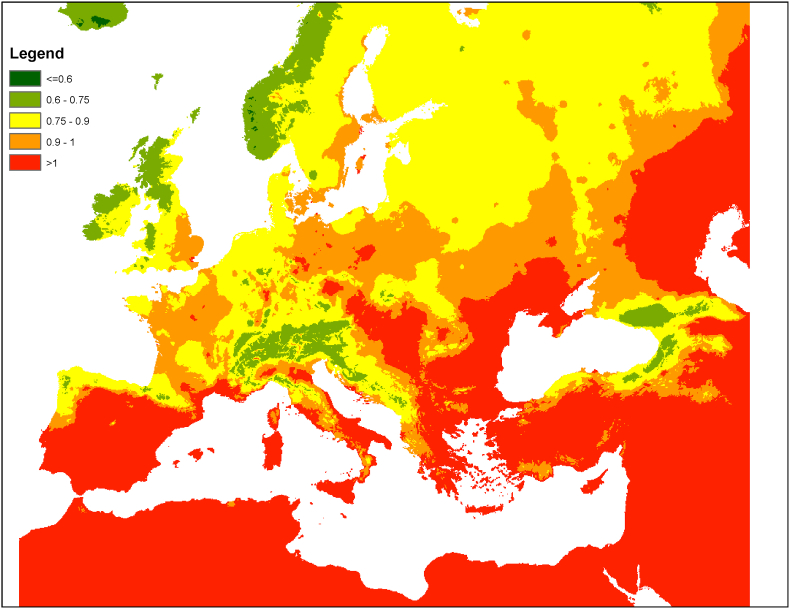
- An example map of the potential water multiplier triggered by desalination in the Mediterranean, under current climate. The water-multiplier represents the long-term additional terrestrial precipitation generated by a steady unit of additional terrestrial evapotranspiration, and is computed here as $\mu =\left(\sum_{i=1}^{\infty }\alpha^{i}\beta^{i-1}\right)$ where α=fraction of terrestrial evapotranspiration that falls back as terrestrial precipitation, and β the fraction of precipitation that is evapo-transpired. The map is derived from β computed with a Budyko model as explained in Pistocchi et al., (2019), assuming the global average value α = 0.57 (van der Ent et al., 2010), and trimming the series in the equation for μ at i=10.

Desalination not only increasingly helps meet a substantial share of water demand and reduce the stress caused by over-abstraction: its recycling in the environment after first use also contributes to plant growth and rainfall. Available estimates (van der Ent et al., 2010) suggest that one m^3^ of plant transpiration yields on average 570 liters of rainfall on land (i.e. an evapotranspiration recycling rate of 57%). We may assume that evaporation of water added to the water cycle through desalination is subject to the same recycling rate (see Supplementary Material, Note 1, for additional discussion). Subsequent cycles of evaporation and precipitation yield a total incremental precipitation inversely related to the ratio between annual runoff and precipitation (Savenije, 1996). This incremental precipitation can be estimated between about 800 (when runoff is 50% of precipitation) and 1300 liters per m^3^ (when runoff is 0%) at global average evapotranspiration recycling rates (see Fig. 1). Precipitation of evaporated water occurs in regions downwind, usually at distances of hundreds to thousands of kilometers (Eltahir and Bras, 1996; Savenije, 1995, Van der Ent and Savenije, 2011). The limited local benefits of evaporation sometimes make it a net loss for a watershed, and measures such as afforestation can impinge on local water resources (Ellison et al., 2012). When these are limited, the positive effect of increased evapotranspiration on atmospheric moisture supply downwind may outweigh the negative local effect of resource depletion. The trade-offs are transboundary in nature (Keys and Ebbesson, 2017). However, when freshwater from desalination is reused for irrigation of forests and agricultural crops in an upwind region, it may contribute to enhancing precipitation downwind without subtracting local natural freshwater. While helping reduce extreme temperatures (Hauser et al., 2019), it would also reduce soil salinization compared with conventional treated wastewater, due to its lower mineral content (Raveh and Ben-Gal, 2018).

Several regions in the world critically depend for their water supply on evapotranspiration upwind (Keys et al., 2012). For instance, central Asia receives rainfall from water evaporated in the Eastern Mediterranean and Gulf Region; the India-Pakistan border from India; the Eastern Sahel from the Mediterranean, etc. Evaporation can also support precipitation in the same region, particularly when meso-scale processes enhance local effects. It has been shown how moisture loading of sea breezes via land evapotranspiration may be critical to trigger condensation and convective precipitation up to about 100 km upstream in the Mediterranean (Millán, 2014; Millán et al., 2005), hence ultimately to support inland freshwater flows. A high-resolution modelling study found that 55% of the evaporation in the Mediterranean is recycled into precipitation in the same region (Jin et al., 2011). Reduced atmospheric moisture supply due to climate change, urbanization and other land cover changes may already be a cause of alteration of the water cycle in this region (Giorgi, 2006; Verdier and Viollet, 2015; Garcia Ruiz et al., 2011; Pausas and Millán, 2019), which can be expected to worsen in the future. Desalination coupled with water reuse for irrigation may reverse this trend.

## 3 Desalination can be fully decarbonized

The energy demand for desalination has been significantly reduced in the last decades and is now as low as 3 kWh/m^3^ (Voutchkov, 2018) or less. As we approach the thermodynamic limit, further energy efficiency gains require more investment (Cohen et al., 2017; Semiat et al., 2010; Shrivastava et al., 2015), and attention to all aspects of plant design where the interaction between different components becomes key to achieving energy efficiency (Karabelas et al., 2018). The source of energy currently used for desalination is very often non-renewable, with a high carbon footprint. The expected growth of desalination, if not coupled with renewable energy (RE), causes a projected 180% increase of carbon emissions by 2040 (Global Clean Water Desalination Alliance, 2015). On the other hand, achieving net-zero greenhouse gas emissions by mid-century (IPCC, 2018) implies covering 100% of energy generation with RE (Bogdanov et al., 2019; Hansen et al., 2019): a strong demand-pull to develop RE-based desalination.

When the power grid can buffer RE generation and desalination energy demand, plants can operate on a continuous basis, and their total energy use can be offset with RE. However, when this is not the case, continuous operation is more difficult.

Technically, the operation of desalination plants may be intermittent, e.g. when powered with battery-less solar PV. On-grid desalination plants could also absorb excess RE produced elsewhere. In this way, while contributing to grid stabilization and flexibility, they would benefit from potentially cheap (or even free) renewable energy. Still, intermittent operation requires buffering the excess water production to meet a less intermittent demand, and implies a trade-off between lower costs of energy and higher capital costs.

The high capital costs of desalination plants, though, suggest energy buffering is often a better option (Caldera and Breyer, 2018).. Today’s battery costs are still high (Freyberg, 2018), a reason for 100% RES-based desalination plants to be still confined mainly to small scale and remote applications (Freyberg, 2018; IRENA, 2012; Cipollina et al., 2015; Brian Publicover, 2019). However, prices have decreased considerably, and are projected to decrease further (Brian Publicover, 2019; Lazard, 2018a, Lazard, 2018b; Schmidt et al., 2017; Vartiainen et al., 2019); in parallel, large scale battery projects increased (Jansen, 2019). Also the costs of PV and onshore wind power plants have decreased in the last years (Jansen, 2019), and RE account now for about 1/3 of the global power capacity (Bellini, 2019; IRENA, 2019), becoming cheaper than nonrenewable energy in a growing number of cases (Lazard, 2018a, Lazard, 2018b). Unsurprisingly, projects of large scale, RE-based desalination are being increasingly developed for industrial (Moore, 2018; Dixon, 2013) as well as domestic water supply (DiPaola, 2019). Estimated costs of hybrid, 100% RE-based desalination systems with battery storage are in the range of 1.00–4.5 €/m^3^ (but mostly below 2.00 €/m^3^) *including* water production and transport (Caldera et al., 2018), close to today’s fossil-fuelled desalination costs (0.60–1.90 €/m^3^ *excluding* the cost of water transport). The modelling of costs (see Supplementary Material, Note 2) of a 100% PV desalination plant scheme with water and energy storage in the Mediterranean (Ganora et al., 2019) highlights energy storage, in whichever form, as more convenient than intermittent plant operation. The most convenient option obviously remains exchanging electricity with the grid: this is not just a key asset to achieve carbon neutrality in general (Child et al., 2019), but also a tool to specifically reduce desalination costs.

## 4 Brine disposal is a manageable issue

According to a recent estimate (Jones et al., 2019), the currently installed global capacity produces about 1.5 m^3^ of brine per m^3^ of desalinated water, causing technical hurdles when (in about 20% of the cases) brine is generated far from the coast. In these cases, minimizing liquid waste discharges is definitely desirable. In the other cases, brine is typically disposed in the oceans. The chemical content of brine depends on the substances added during desalination and on the release of contaminants from the desalination equipment. While this can sometimes be an issue (Roberts et al., 2010), contamination can be prevented through appropriate plant design (Gude, 2016). Brine disposal may affect marine ecosystems (Lattemann, 2010), and particularly primary producers such as seagrass meadows (Garcia et al., 2007; Fernández-Torquemada and Sánchez-Lizaso, 2011), and benthic communities (de-la-Ossa-Carretero et al., 2016) if not adequately diluted. A strong dilution of brine usually occurs near its sea outfall, particularly if velocities are high. After this initial dilution, higher-salinity density currents are formed that propagate over hundreds to thousands of meters with limited additional dilution (Pistocchi et al., 2020). Therefore, adequate dilution in the near field is key to ensure control of impacts. This can be achieved with appropriate design of outfalls, and particularly outfall water velocity. Brine dilution can be further enhanced by mixing with cooling water from power plants (e.g. at plants in Ashkelon and Hadera, Israel, each of capacity >120 million m^3^/year: Miller et al., 2015) or other less concentrated streams. When this is the case, the distance at which dilution is sufficient can be reduced to a few tens of meters (Bleninger and Jirka, 2008). In the 100 million m^3^/year capacity plant of Tuas Spring (Singapore), the intake is only about 150 m from the brine discharge, proving negligible impact on salinity at that distance. If the whole coastal population of the Mediterranean were serviced by 200 plants the size of Tuas Spring, we would expect 200 impacted regions of 150 m radius, i.e. less than 15 km^2^, or 0.03% of a 1-km wide buffer of the about 46,000 km long Mediterranean coastline). Recent studies (Wood et al., 2020; Clark et al., 2018) suggest that a high water velocity, hence dilution at the point of brine discharge may cause hydrodynamic disturbance, as well as more persistent density currents in the far field, with potentially negative ecological implications, prompting the appraisal of brine disposal on a case-by-case basis.

The sea disposal of smaller volumes of more concentrated brine could also reduce the costs of disposal, although it would add to the challenge of meeting the initial brine dilution requirements (Pistocchi et al., 2020). In principle, brine may be concentrated up to saturation in salts, ∼10 times seawater salt concentration, allowing recovery of ∼80% of the its water content.. To this end, passive solar evaporation could produce water vapour useful for cooling and humidifying specifically designed “seawater greenhouses” representing a sustainable option for agriculture in arid climates (Akinaga et al., 2018). In many cases, though, evaporative concentration of the brine entails large areas of evaporation ponds or significant energy input, hence costs.

## 5 Is resource recovery a game changer for desalination?

**Fig. 2:**
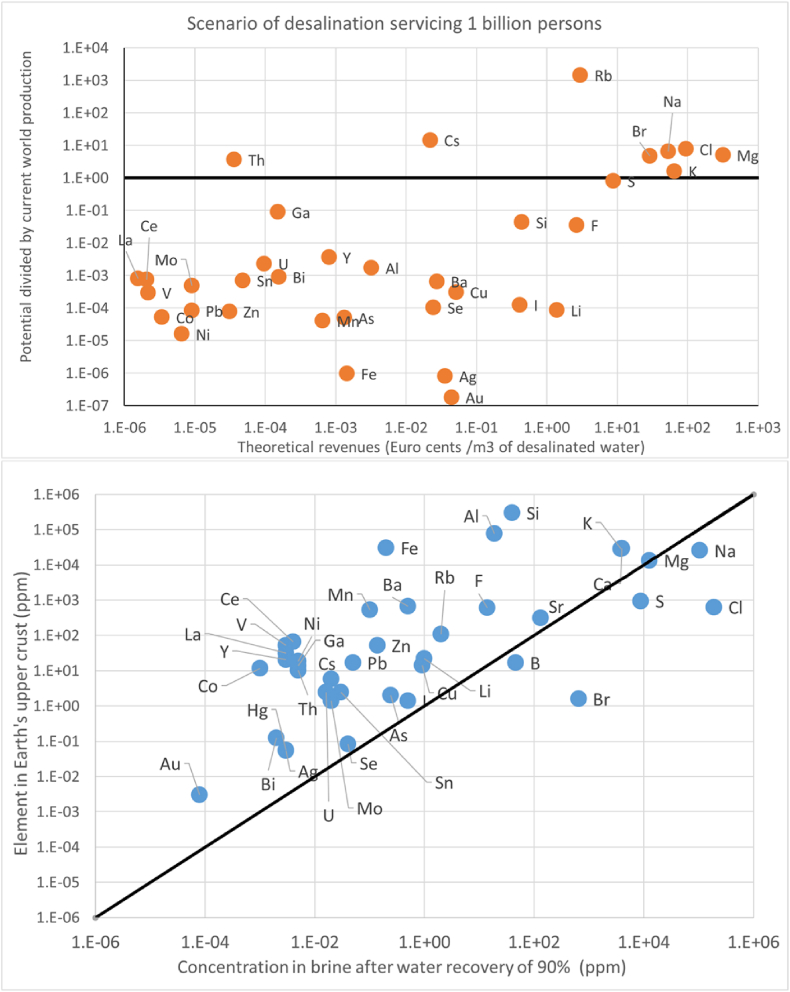
Upper pane: potential revenue from selling a mineral at current price, vs ratio of potential production to current global demand. Lower pane: comparison of average concentrations in seawater and upper Earth crust (with 1:1 line). Data details and sources are in Supplementary Material, Note 3.

Concentrated brine could also facilitate the recovery of valuable minerals from seawater, or “seawater mining”, sometimes advocated as a possible way to offset the costs of desalination (e.g. Diallo et al., 2015, Quist-Jensen et al., 2016; Loganathan et al., 2017). We compare the revenues one could expect at current prices, and the quantities one could obtain from desalination servicing 1 billion people with 200 L person^−1^day^−1^. Fig. 2 (see also Supplementary Material, Note 3) shows potential candidate elements usually having high-value and very low concentration (e.g. Au, Ag), low concentration and intermediate value (e.g. Cu, Li), high concentration and low value (e.g. sea salt), and relatively high value and concentration (Mg, K and Br). The theoretical revenue expected from selling the minerals contained in seawater is < 0.1 € m^−3^ for Li, F, S, Rb, <0.01 € m^−3^ for I and Si, and <0.001 € m^−3^ for all other minerals. Only sea salt (NaCl), Br, Mg and K yield higher revenues. If salt production were implemented universally, the amount produced would exceed the total global demand by a factor ∼10, making either the production impossible to sell, or prices to fall. The production seems excessive even considering increased demand due to new applications, e.g. in refrigeration through solar liquid desiccation (Lychnos et al., 2012). Similar considerations apply to Br and Mg, although the latter may have a higher economic interest. Mineral recovery could be justified at specific desalination plants, where the logistics foster access to markets. In these cases, revenues could even be high enough to offset desalination costs. More general could be the opportunity for recovery of K, whose availability in seawater could match the global demand of this element for fertilizers and other applications (Manning, 2015; Ciceri et al., 2015). However, technologies to economically separate K from seawater are not yet available (Ciceri et al., 2015). Mineral recovery remains difficult to generalize. It may gain some traction with the development of separation technologies, as land reserves dwindle, or because of escalating social or geopolitical tensions. This is particularly true for minerals whose abundance in seawater is comparable with, or higher than their average abundance in the upper Earth crust (see Fig. 2). In such scenarios, we may expect that certain minerals could become profitable by-products of desalination, but the expected revenues would only marginally affect the cost of desalinated water. Thus, while an intriguing challenge for the future, seawater mining is currently hardly a game changer for desalination.

Attempts at harnessing the chemical energy embedded in seawater rely at present mainly on pressure-retarded osmosis (PRO) and reverse electro-dialysis (RED) (Yip et al., 2016). PRO consists of putting a low salinity feed solution in contact with a pressurized, higher salinity draw solution through a semi-permeable membrane (Helfer et al., 2014). Even with high salinity gradients, PRO can only contribute a small fraction of the energy required for desalination, as we show in the Supplementary Material, Note 4. While RED is experiencing developments (e.g. Tedesco et al., 2017), it can similarly marginally offset the energy demand of desalination.

In PRO, the brine is mixed with lower concentration water such as seawater. Therefore, when pre-dilution is necessary to meet the initial dilution requirements of brine, this method of energy recovery may become attractive even with relatively poor energy performances not justifying an investment for RE generation alone.

## 6 Desalination costs can be affordable when properly managed

Desalination costs still seem excessive in less developed economies (e.g., Gude, 2016). A recent global assessment (Gao et al., 2017) suggests that desalination would become economically feasible in countries undergoing continued development by 2050, as a combined outcome of cost reduction, water scarcity and increasing water prices.

Adequate water pricing is prerequisite to secure the large investments required by desalination, and water pricing requires a stable water governance. As such, desalination is likely to emerge more because of, rather than as a tool for, regional cohesion and political stability. Broadly augmenting natural processes with a technological solution for water supply may lead to a strong commodification of this essential resource. On the other hand, a sound water pricing system can also cater for financial margins allowing socially equitable tariffs, hence supporting universal access to water according to the sustainable development goals. This in turn may help quench social and geopolitical conflicts on water.

Although in some economies water may be freely accessible, most often it comes at a price, if only the resources spent to access remote water sources (Nastiti et al., 2017). For instance, the cost of manual abstraction of freshwater from a remote source, as still happens in many parts of the world, is arguably much higher than the cost of desalination.. Households in developing countries support hidden costs for unreliable water services, and would be willing to pay more for having them more reliable (e.g. Pattanayak et al., 2005; Anteneh et al., 2019; Semiat, 2008). Often, because water is considered a basic right, our societies warrant access to water at subsidized prices (e.g. Dugard et al., 2017) or through appropriate regulation (e.g. Pezon, 2017), although measures adopted to this end are usually not well documented and understood (Fuente and Bartram, 2018). As a consequence, the costs of water are often borne by the society through taxes, transfers or cross-subsidization, or by accepting a certain level of stress in aquatic ecosystem in order to reduce the direct monetary price paid for water. As desalinated water could be equally subsidized, its costs should not be compared with the production costs of conventional drinking water alone, but with its full costs: while desalination usually has a higher production cost than conventional resources, it has virtually no environmental cost if energy is decarbonized and brine disposal is appropriate. On the contrary, the cost of conventional resources abstractions in terms of water stress on ecosystems is seldom factored in (e.g. Korsgaard and Schou, 2009; López-Morales, 2017). Finally, while costs of desalination decrease (Cohen et al., 2017; Ghaffour et al., 2013), costs of conventional water resources increase as they become less accessible (e.g., 300 m deep groundwater pumping requires about 1 kWh/m^3^), or require treatment for pollution, making costs more similar even at face value.

Arguably, the price of desalinated water reflects its full economic value explicitly. This price signal may stimulate higher value-added applications: for instance, direct or indirect use (reuse) of desalinated water for irrigation is justified only by a productive and profitable cropping model (Garcia-Caparros et al., 2017; Sanchez, 2015) also entailing more efficient irrigation (Frija et al., 2011; Rodríguez-Ferrero et al., 2010). If properly managed, desalination could be a flywheel for development, with minimal negative social impacts: particularly, desalination-based agriculture could develop in regions where more traditional production systems could not survive.

In order to maximise its full benefits, desalination should be developed using technologies and design concepts specifically fitting the context. Economies of scale are an important push for centralized, large scale plants with high efficiency and recovery (possibly including brine resources recycling in some cases). Yet, specific local conditions may make a full array of thermal, electrochemical and membrane solutions preferable at small scale (depending on the connection to the power grid, the presence of users of desalination by-products, low-cost energy sources such as waste heat, etc.). In this perspective, large plants may coexist with plants at lower efficiency, but also lower cost and impact, avoiding a “one-size-fits-all” approach.

## 7 Conclusions

- Desalination can be a sustainable way to replenish our water cycle: after primary uses (industrial or domestic), reuse of desalinated water for irrigation enables agriculture in otherwise unproductive regions, and/or forest growth. The ensuing evapotranspiration feeds the water cycle, further enhancing precipitation, while enabling carbon sequestration by plants. In this way, desalination may not only reduce freshwater abstraction, but also provide a net water surplus, and thus help preserve and restore freshwater-dependent ecosystems.
- The negative effects of desalination can be effectively controlled. Energy efficient desalination is now ready to harness renewable energy sources (RE), particularly photovoltaics (PV) combined with battery storage, in increasingly competitive ways, so as to become carbon-neutral. Brine disposal affects marine ecosystems only at local scale, and appropriate design of outfalls can minimize impacts through dilution.
- Resource recovery from brine is of marginal help with current technologies, in spite of the appreciable content of valuable minerals and energy, because their concentration is so low that revenues from their recovery cannot offset the costs of water production. Yet, when operated with cheap energy (e.g. using passive solar evaporation or waste heat) concentration of brine may yield additional water resources while reducing the costs of sea disposal.
- While carbon-neutral desalination can already be affordable, it may become mainstream as societies understand its broader benefits. With market expansion, costs will decrease further, triggering a virtuous cycle of water resources replenishment. For this scenario to come true, desalination cannot work in isolation, but through integrated water management including reuse for irrigation, maximizing the social and environmental return.

## Declaration of competing interest

Nothing to declare.
