# Supplementary material for: Can seawater desalination be a win-win fix to our water cycle?
Source: Water Res. 2020 Sep 1;182:115906. doi: 10.1016/j.watres.2020.115906 (PMC7487278; doi:10.1016/j.watres.2020.115906)
Supplement: Multimedia component 1 [file mmc1.docx]

Making waves:

Can seawater desalination be a win-win fix to our water cycle?

A.Pistocchi^[[1]](#footnote-1)^*, T.Bleninger^[[2]](#footnote-2)^, C.Breyer^[[3]](#footnote-3)^, U.Caldera^3^, C.Dorati^1^, D.Ganora^[[4]](#footnote-4)^, M.M.Millan^[[5]](#footnote-5)^, C.Paton^[[6]](#footnote-6)^, D.Poullis^[[7]](#footnote-7)^, F.Salas Herrero^1^, M.Sapiano^[[8]](#footnote-8)^, RSemiat^[[9]](#footnote-9)^, C.Sommariva^[[10]](#footnote-10)^, S.Yuece^[[11]](#footnote-11)^, G.Zaragoza^[[12]](#footnote-12)^

# Supplementary material

f

## Note 1

It has been observed that irrigation not only increases moisture supply, but may also affect atmospheric circulation, reducing moisture transport. For instance, Ter Maat et al. [10] simulate the change in precipitation over southwestern Saudi Arabia due to a large irrigation expansion and conclude that the additional atmospheric moisture supply does not yield a proportional precipitation increase, because of the simultaneously weakened sea breezes. Puma and Cook [11], in their analysis of the effects of irrigation on the global climate of the XX century, find both an increase in precipitation in many regions downwind of irrigation, and a weakening of the Indian monsoon. However, irrigation’s positive effect of increasing moisture supply often outweighs its downside of circulation weakening: for instance Pei et al. [12], while finding slightly reduced precipitation over irrigated areas, evaluate a significantly enhanced precipitation in regions downwind; Kang and Eltahir [13] show how increased precipitation and surface cooling may be attributed to irrigation in the North China Plains. In the Mediterranean region, conversely, Pausas and Millan [5] attribute the dwindling of summer convective precipitation to the drastic reduction of moisture supply near the coast, due to the replacement of irrigated agriculture and wetlands with dry urban surfaces.

Irrigation conveys water to plants that transpire it to the atmosphere. The evapo-transpiration rate depends on the type of plants, and is usually found to be highest for forests [e.g., 2]. Therefore, the most efficient way to inject additional moisture to the water cycle is arguably the irrigation of forests. More fundamentally, forests have been described as a “biotic pump” [3, 4] that, by evaporating water from the plants, reduces the precipitation returning to the sea as runoff and recycles it as rainfall inland. In this way forests are key to transporting water inland from coastal areas through cycles of evapotranspiration and precipitation. As such, forests have been defined a global public good [1] because of the planetary scale of their beneficial effects. In this framework, the expansion of vegetation is key for the regulation of water availability and climate [7]. Afforestation could be a strategy to increase precipitation by inducing moisture recycling, although the use of freshwater to irrigate new vegetation has been flagged as possibly competing with other water uses [6]. Recycling desalinated water through irrigation after its first use, instead of wasting it through disposal, may support afforestation in otherwise unusable land such as desert margins, without such competition. The increased evapotranspiration not only enhances the water cycle in drylands, but reduces local temperatures while increasing carbon sequestration [7]. Desalination-supported, large-scale afforestation of deserts has been boldly suggested as a priority for the mitigation of climate change [8], with a widespread potential for tree restoration in many water-stressed regions of the world for a total of almost 1 billion additional hectares of canopy cover estimated globally [9].

## Note 2

The concept of a 100% PV-based desalination plant with a modular scheduling of water production following the monthly variability of radiation, battery and water storage has been proposed to increase autonomy from the grid in the extended Mediterranean region [17]: a saltwater reservoir at a certain elevation, followed by a booster pump, enables splitting the "fixed" energy demand of membrane operation from the "flexible" demand for pumping to the reservoir, which may use PV power as it is available. For plant CAPEX and OPEX we consider the range of costs from [18], including Intake construction, pretreatment, construction, RO system equipment, post-treatment, waste handling (membrane cleanup), waste handling (open intakes backwash), electrical/instrumentation systems, auxiliary costs, building costs, startup/commissioning/acceptance testing, design, construction management and pilot testing, administration, environmental permitting and legal. The sum of these costs ranges from $1,255.00 to $4,315.00 per m^3^ day^-1^ of plant capacity. We assume the upper bound to correspond to a small plant servicing 1,000 persons, and the lower bound to a large plant servicing 300,000 persons. We interpolate the two values with a power law, resulting in the following expenditure function:

$$C_{CAPEX}=19254P^{-0.217}$$

where $C_{CAPEX}$ is the investment cost of a RO desalination plant ($ m^-3^ day) and P the serviced population (count). Given the indicative purpose of the calculation, and in order to account for the price increases occurred since [18], we make US dollars to coincide with Euro, hence $C_{CAPEX}$ is meant in € m^-3^ day *tout court*.

For operation and maintenance (O&M), [18] provides a range of 0.1 to 0.27 $ m^-3^ including maintenance, parts replacement, waste disposal, monitoring, and indirect costs. As above, we assume the upper bound to correspond to a small plant servicing 1,000 persons, and the lower bound to a large plant servicing 300,000 persons. We interpolate the two values with a power law, resulting in the following expenditure function:

$$C_{OPEX}=0.899P^{-0.174}$$

where $C_{OPEX}$ is the O&M cost of a RO desalination plant ($ m^-3^) and P the serviced population (count). Also in this case, we assume Euro and US dollars to coincide. The potentially serviced population is estimated as the one reached with desalinated water transport cost estimated below 0.5 $ m^-3^ (See [19]).

We assume the cost of treatment to coincide with the cost of energy required for RO filtration, hence:

$$C_{T}= C_{PV}\varepsilon$$

where $\varepsilon$ is the specific energy requirement of RO desalination (kWh m^-3^) and $C_{PV}$ is the levelized cost of photovoltaic energy (€ kWh^-1^) as discussed in [19].

The cost of electric batteries is computed as $C_{B}=250 \beta$, where $\beta$ is the battery capacity requirement and the cost of batteries is assumed equal to 250 Euro kWh^-1^; the cost of water reservoirs is similarly computed as $C_{R}=65 \rho$, where $\rho$ is the reservoir capacity requirement and the cost of water storage is assumed equal to 65 Euro m^-3^. We assume O&M costs of both batteries and reservoirs to be implicitly incorporated in the above figures.

In order to convert the investment costs (referred to capacity) to levelized costs (referred to the unit of produced water), we make use of the “present value of annuity” factor:

$pva\left( r,n \right)=\frac{1-\left( \frac{1}{1+r} \right)^{n}}{r}$,

where r is the annual interest rate and n is the number of years of useful life (or depreciation period) of the investment. We assume r=5% and n=5 for batteries, n=20 for the desalination plant and n=50 for reservoirs. The total levelized cost of desalinated water is computed as:

$LCoW=C_{T}+C_{OPEX}+\frac{C_{CAPEX}}{365 pva(0.05, 20)}+\frac{C_{B}}{365 pva(0.05, 5)}+\frac{C_{R}}{365 pva(0.05, 50)}$.

For batteries, we consider also a more optimistic scenario where n=10 and $C_{B}=125 \beta$. The analysis presented in [17] yields parameters β and ρ as well as the energy exchanged with the grid under various scenarios. In order to estimate the costs presented in Figure SM1, we consider for each strategy the most optimistic, as well as the bottom line conditions.

The costs of this scheme are higher in comparison with the case where electricity is fed to, or withdrawn from the grid so to ensure continuous operation (Figure SM1), but are lower compared to those when the plant would operate intermittently (coupled with PV energy). The case where energy is stored in batteries alone or as a complement to the water reservoir is somewhere in between (Figure SM1). However, simple battery storage might become the most convenient option if battery capital cost decreases and the cost of power exchange with the grid increases (Figure SM1).

*
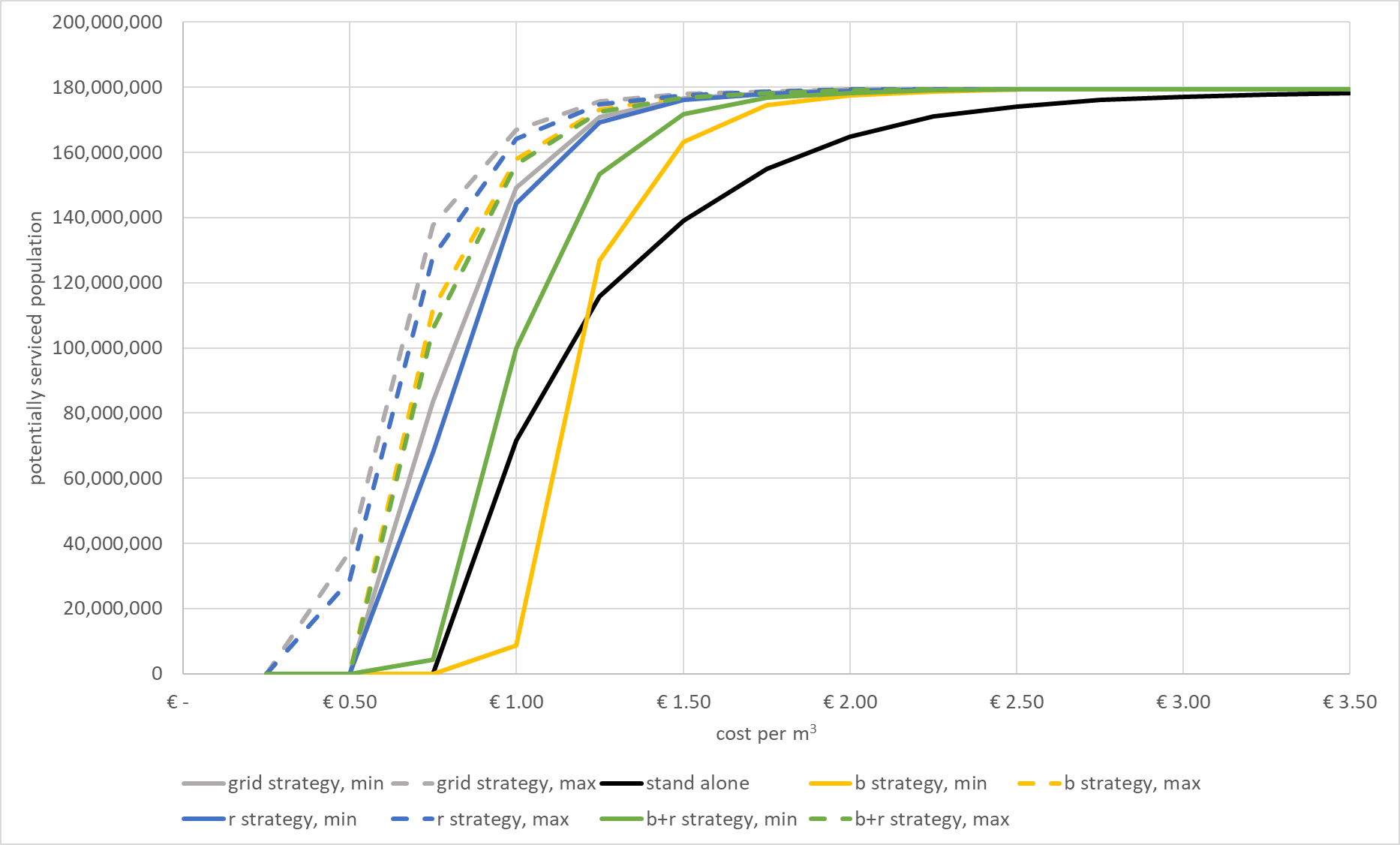

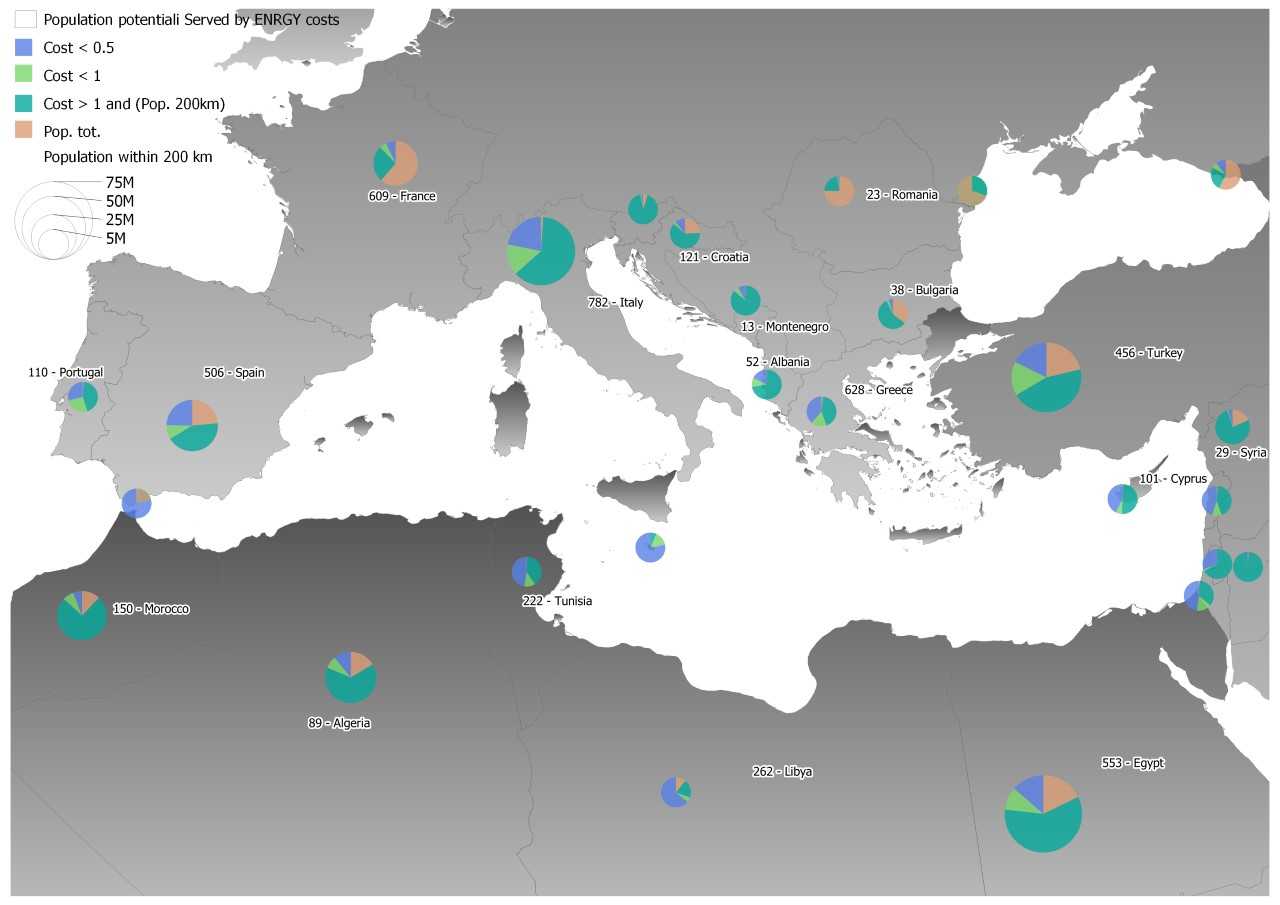
*

*Figure SM1 – Below: population that can be reached at different transport costs (0.5, 1 and > 1 €/m^3^). Above: costs of water production under different assumptions in the Mediterranean . The different strategies for energy storage are: grid= only exchange with the grid; b=battery; r=water reservoir; b+r=combined reservoir and battery. "Min" and "Max" correspond to combinations with cost of power exchange with the grid of 1and 10 Euro Cents/kWh; for battery and reservoir, we consider both an optimistic and a bottom line scenario whereby battery cost is higher and battery life is shorter.*

## Note 3

*Table SM 1 – minerals in seawater*

We provide a compilation of data on minerals in seawater. It should be noted that prices are purely indicative, highly variable and uncertain. For prices, we could not find a single, homogeneous source of information, and in many cases we compiled evidence available on intelligence or business web sites.

| **Mineral** | **Conc. in seawater (ppm)^[[13]](#footnote-13)^** | **Conc. in continental crust (ppm)^[[14]](#footnote-14)^** | **price Eur/tonne** | **source** | **world production tonnes/year^[[15]](#footnote-15)^** |
| --- | --- | --- | --- | --- | --- |
| Cl | 18980 | 640 | € 50 | indicative price assumed | 180,000,000 |
| Na | 10561 | 25670 | € 50 | indicative price assumed | 120,000,000 |
| Mg | 1272 | 13510 | € 2,500 | <https://twitter.com/CasperBurgering/status/1019898064348372992> | 18,370,000 |
| S | 884 | 953 | € 100 | <https://www.icis.com/explore/resources/news/2019/07/19/10393885/global-sulphur-prices-weaken-further-on-high-stocks-weak-demand> | 80,000,000 |
| K | 380 | 28650 | € 1,707 | <https://rmis.jrc.ec.europa.eu/uploads/rmprofiles/Potash.pdf> | 17,220,000 |
| Br | 65 | 1.6 | € 4,500 | https://www.japanchemicaldaily.com/2018/01/03/bromine-price-record-high-chinese-market/ | 1,000,000 |
| Si | 4 | 303480 | € 111,111 | <https://www.radiochemistry.org/periodictable/elements/14.html> | 6,700,000 |
| Al | 1.9 | 77440 | € 1,700 | <https://www.lme.com/Metals/Non-ferrous/Aluminium#tabIndex=0> | 80,300,000 |
| F | 1.4 | 611 | € 1,900,000 | <https://www.chemicool.com/elements/fluorine.html> | 2,842,000 |
| Rb | 0.2 | 110 | € 15,000,000 | USGS Mineral commodities summary 2019 (Rb 100 g ampoules) | 10 ^16^ |
| Li | 0.1 | 22 | € 140,000 | <https://twitter.com/CasperBurgering/status/1019898064348372992> | 85,000,000 |
| Cu | 0.09 | 14.3 | € 5,700 | <https://www.lme.com/Metals/Non-ferrous/Copper#tabIndex=0> | 21,000,000 |
| Ba | 0.05 | 668 | € 550,000 | <https://www.chemicool.com/elements/barium.html> | 5,510,000 |
| I | 0.05 | 1.4 | € 83,000 | <https://www.chemicool.com/elements/iodine.html> | 29,000,000 |
| As | 0.024 | 2 | € 556 | <https://www.joc.com/spot-metals-prices_19990825.html> | 35,000,000 |
| Fe | 0.02 | 30890 | € 72,000 | <https://www.chemicool.com/elements/iron.html> | 1,500,000,000 |
| Zn | 0.014 | 52 | € 2,200 | <https://www.lme.com/Metals/Non-ferrous/Nickel#tabIndex=0> | 13,000,000 |
| Mn | 0.01 | 527 | € 65,000 | <https://www.chemicool.com/elements/manganese.html> | 18,000,000 |
| Pb | 0.005 | 17 | € 1,800 | <https://www.lme.com/Metals/Non-ferrous/Lead#tabIndex=0> | 4,400,000 |
| Se | 0.004 | 0.083 | € 61,000 | <https://www.chemicool.com/elements/selenium.html> | 2,800,000 |
| Sn | 0.003 | 2.5 | € 16,000 | <https://www.lme.com/Metals/Non-ferrous/Tin#tabIndex=0> | 310,000 |
| Cs | 0.002 | 5.8 | € 11,000,000 | <https://www.chemicool.com/elements/cesium.html> | 10 ^[[16]](#footnote-16)^ |
| Mo | 0.002 | 1.4 | € 4,500 | <https://www.lme.com/Metals/Minor-metals/Molybdenum-Platts#tabIndex=0> | 300,000 |
| U | 0.0016 | 2.5 | € 60,000 | indicative price assumed | 50,000 |
| Ga | 0.0005 | 14 | € 300,000 | <http://strategic-metal.com/products/gallium/gallium-price/> | 410 |
| Ni | 0.0005 | 18.6 | € 13,000 | <https://www.lme.com/en-GB/Metals/Non-ferrous/Nickel#tabIndex=0> | 2,300,000 |
| Th | 0.0005 | 10.3 | € 72,000 | USGS Mineral commodities summary 2019 (price of import to US from India) | 10^16^ |
| Ce | 0.0004 | 65.7 | € 5,180 | assumed equal to La | 38,000 ^[[17]](#footnote-17)^ |
| V | 0.0003 | 53 | € 7,200 | <https://ukinvestormagazine.co.uk/ferro-alloy-shares-crash-on-falling-vanadium-prices/> | 73,000 |
| La | 0.0003 | 32.3 | € 5,180 | <https://www.statista.com/statistics/450139/global-reo-lanthanum-oxide-price-forecast/> | 27,000 ^17^ |
| Y | 0.0003 | 20.7 | € 2,678,571 | <http://chemistry.pomona.edu/Chemistry/periodic_table/Elements/Yttrium/yttrium.htm> | 6,000 |
| Ag | 0.0003 | 0.055 | € 1,200,000 | <https://www.chemicool.com/elements/silver.html> | 27,000,000 |
| Bi | 0.0002 | 0.123 | € 780,000 | <http://chemistry.pomona.edu/Chemistry/periodic_table/Elements/Bismuth/bismuth.htm> | 16,000 |
| Co | 0.0001 | 11.6 | € 34,000 | <https://www.lme.com/Metals/Minor-metals/Cobalt#tabIndex=0> | 140,000 |
| Au | 0.000008 | 0.003 | € 55,400,000 | <https://www.chemicool.com/elements/gold.html> | 3,200,000 |

## Note 4

In this contribution we focus on PRO.

The power density through the membrane (W m^-2^) potentially available from PRO can be represented as [15, 16]:

$$W=A\left( \xi\Pi-P \right) P$$

where P is the osmosis-retarding pressure applied to the draw solution, A is the membrane permeability coefficient, and $\Pi$ is the osmotic pressure difference between the bulk of the feed and the bulk of the draw solutions before they mix. The coefficient $\xi<1$ accounts for the reduced average osmotic pressure difference across the membrane due to concentration polarization and salts back-diffusion effects, and for the concentration (hence, osmotic pressure) gradient reduction with the progress of permeation of the feed solution along the membrane. A justification of the above equation is presented below.

The power density (W m^-2^) that can be obtained from PRO is usually presented as [15, 16]:

$$W=J P$$

where P is the osmosis-retarding pressure applied to the draw solution and J is the water flux through the membrane. P is generally designed as the pressure maximizing W, where J is calculated as [15, 16]:

$$J=A\left( \hat{\Pi}-P \right)$$

where A is the membrane permeability coefficient, $\hat{\Pi}$ is the average osmotic pressure difference across the membrane skin. This is always lower than the osmotic pressure difference between the bulk of the feed and the bulk of the draw solutions, because of concentration polarization (CP) and salts back-diffusion (SBD) effects. Moreover, the permeation of the feed solution along the membrane causes the concentration, hence osmotic pressure gradient to decrease. Along a membrane of length L, at a given abscissa $x$, we will have a local osmotic pressure difference between the faces of the membrane equal to:

$$\pi\left( x \right)=\pi^{bulk}(x)\varphi(x)$$

where $\pi^{bulk}\left( x \right)$ is the local osmotic pressure difference between the bulk of the feed and draw solutions, and $\varphi(x)$ < 1 is a function representing the effect of CP and SBD. In turn, we may write

$$\pi^{bulk}\left( x \right)=\Pi\omega(x)$$

where $\Pi$ is the osmotic pressure difference between the draw and feed solutions before they are put in communication through the membrane, and $\omega(x)$<1 is a function representing the effect of dilution of the draw solution as permeation progresses. If we posit:

$$\xi= \frac{1}{L}\int_{0}^{L} \omega(x)\varphi(x)dx$$

the power density through the membrane can be written as:

$$W=A\left( \xi\Pi-P \right) P$$

While Yip et al. [15] propose an analytical equation for $\pi(x)$, Sagiv et al. [14] compute $\varphi(x)$ and $\omega(x)$ implicitly using a CFD model for a membrane module of L=1 m. These Authors consider $\Pi$ corresponding to combinations of (1) Dead Sea water (5.8 M) and seawater brine (1 M), (2) seawater (0.6 M) and brackish water (0.01 M), and (3) seawater brine and brackish water. Their analysis suggests $\xi$ to be 0.035, 0.329 and 0.425 respectively for these three combinations (See Table A3 in [14]).

The net power density of a PRO membrane is:

$$W_{n}=A\left( \xi\Pi-P \right)\left( P-P_{loss} \right)\eta_{T}-q \left( \frac{1}{\eta_{P}}-\eta_{T} \right)P-q P_{loss}\eta_{T}-w'$$

where $P_{loss}$ are frictional pressure losses in the draw solution channel, $\eta_{T}$ and $\eta_{P}$ are the efficiencies of energy recovery device (e.g. turbine) and pump respectively, q is the flow of draw solution through the plant and w’ is power required to overcome frictional losses in the feed solution channel. By imposing $\frac{\partial W_{n}}{\partial P}=0$, we find the value of P that maximizes W_n_:

$$P=\frac{\xi\Pi+P_{loss}-\frac{q}{A}\left( \frac{1}{\eta_{P}\eta_{T}}-1 \right)}{2}$$

This value is usually quite lower than the pressure maximizing W_n_ in the absence of friction and other energy losses, i.e. $\frac{\Pi}{2}$. The real power density of a PRO plant, W_n_, must be evaluated taking into account the efficiency of the energy recovery devices (ERD) as well as the power required to pump the feed and draw solutions through the plant, and pressure losses. Sagiv et al. [14] compute $W_{n}$ numerically for various combinations of feed and draw solutions, membrane properties and equipment efficiencies, and show that obtaining an economically sustainable power density over a 1-m long membrane module requires a pump and ERD efficiency unrealistically close to 100% even for the highest salinity gradient considered in their exploration. Their simulations use a draw solution pressure P rather close to $\frac{\Pi}{2}$ (the draw solution pressure maximizing W for $\xi=1$). With the value of P maximizing $W_{n}$ and the salinity gradient of brine concentrated to 10 times the concentration of seawater as draw solution, coupled with seawater as feed, a sustainable power density may be still obtained using realistic pump and turbine efficiencies (85-90%) as shown in the example calculation of Table SM2. Even under these circumstances, though, the energy produced per cubic meter of brine is rather low (less than 0.4 kWh/m3), and the amount of energy produced per cubic meter of desalinated water is proportionally even smaller (in the order of 0.1 kWh/m3). Taking into account the energy required to concentrate the brine by a factor ~5 (e.g. via evaporation), the energy extractable via PRO does not appear sufficient to significantly reduce the overall desalination energy consumption (hence costs). On the contrary, the expected capital and operation cost of a PRO plant might make the cost of energy rather high compared to alternative RES. These considerations show the limits of the economic case for energy recovery from brine.

*Table SM2 – example calculation of energy production from PRO, assuming* $\xi=0.4$*, membrane and draw/feed solution flow characteristics as in Sagiv et al.[73]. It should be noted that q cannot be reduced below a minimum threshold given by the accumulated amount of permeate along the membrane. In cases where* $\xi<0.4$ *the argument is valid a fortiori, while even assuming* $\xi=1$ *would lead to a production of about 0.2 kWh/m3 of desalinated water, i.e. <10% of the energy required for desalination, excluding brine concentration.*

| **Membrane and system properties** | | |
| --- | --- | --- |
| Pressure for feed lift | 0.00E+00 | Bar |
| Membrane permeability (A) | 3.80E-12 | m s-1 Pa-1 |
| Salt permeability coefficient (B) | 5.10E-07 | m s-1 |
| Energy Recovery Device efficiency | 90.0% | [-] |
| Pump efficiency | 85.0% | [-] |
| Membrane length | 1.00E+00 | m |
| Membrane surface | 1.00E+00 | m2 |
| Feed channel width | 1.00E+00 | m |
| Feed channel height | 4.00E-04 | m |
| feed channel Hydraulic diameter | 2.00E-04 | m |
| Feed cross velocity | 1.04E-01 | m s-1 |
| Feed flow | 4.16E-05 | m3 s-1 |
| Feed Reynolds number | 2.26E+01 | [-] |
| Feed Pressure loss | 6.76E-01 | Bar |
| Draw channel width | 1.00E+00 | m |
| Draw channel height | 4.00E-04 | m |
| draw channel Hydraulic diameter | 1.31E-03 | m |
| Draw cross velocity | 1.18E-01 | m s-1 |
| Draw flow | 2.36E-05 | m3 s-1 |
| Draw Reynolds number | 7.04E+01 | [-] |
| Draw Pressure loss | 2.85E-02 | Bar |
| **Solutions properties** | | |
| draw concentration/seawater concentration | 9.61E+00 | [-] |
| feed concentration/seawater concentration | 1.00E+00 | [-] |
| Seawater salinity | 3.50E+01 | Psu |
| Osmotic pressure draw | 2.59E+02 | Bar |
| Osmotic pressure feed | 2.70E+01 | Bar |
| Osmotic pressure difference | 2.32E+02 | Bar |
| Draw density | 1.24E+03 | Kg m-3 |
| Draw kinematic viscosity | 2.20E-06 | m2 s-1 |
| Feed density | 1.02E+03 | Kg m-3 |
| Feed kinematic viscosity | 9.21E-07 | m2 s-1 |
| **Water flux calculation** | | |
| Reduction factor for osmotic pressure difference due to conc. Polar. | 40.0% | [-] |
| Draw solution pressure | 3.69E+01 | Bar |
| Water flux | 2.13E-05 | m s-1 |
| **Power calculation** | | |
| Theoretical power generation | 2.46E+02 | W m-2 |
| Gross generated power | 7.05E+01 | W m-2 |
| Power used for pumping | 1.06E+02 | W m-2 |
| Power recovered from draw | 7.83E+01 | W m-2 |
| Net generated power | 4.31E+01 | W m-2 |
| membrane surface required | 2.32E+01 | m2 kW-1 |
| energy recovery per m3 of draw solution | 5.07E-01 | kWh m-3 |
| m3 of draw solution per m3 of desalinated water | 2.08E-01 | [-] |
| energy recovery per m3 of desalinated water | 1.06E-01 | kWh m-3 |
| disposed brine salinity | 1.94E+02 | Psu |

References

1. Ellison, D., Futter, M.N., Bishop, K., On the forest cover-water yield debate: from demand- to supply-side thinking. Global change biology, 18, 806-820, 2012
2. L. Zhang, W.R. Dawes, G.R. Walker, Response of mean annual evapotranspiration to vegetation changes at catchment scale. Water Resour. Res., 37 (3) (2001), pp. 701-708
3. Makarieva, A.M., Gorshkov, V.G., Biotic pump of atmospheric moisture as driver of the hydrological cycle on land. Hydrology and Earth System Sciences, 11, 1013-1033, 2007
4. Sheil, D., Forests, atmospheric water and an uncertain future: the new biology of the global water cycle. Forest Ecosystems 5, 19 (2018), <https://doi.org/10.1186/s40663-018-0138-y>
5. Pausas, J. G., Millán, M.M., Greening and Browning in a Climate Change Hotspot: The Mediterranean Basin, BioScience, 69 (2) 2019, pp 143–151, <https://doi.org/10.1093/biosci/biy157>
6. Layton, K., Ellison, D., Induced precipitation recycling (IPR): a proposed concept for increasing precipitation through natural vegetation feedback mechanisms. Ecological engineering, 91, 553-565 (2016)
7. Ellison, D., C.E. Morris, B. Locatelli, D. Sheil, J. Cohen, D. Murdiyarso, V. Gutierrez, M. van Noordwijk, I.F. Creed, J. Pokorny, D. Gaveau, D. V. Spracklen, A. Bargués Tobella, U. Ilstedt, A.J. Teuling, S. G. Gebrehiwot, D. C. Sands, B. Muys, B. Verbist, E. Springgay, Y. Sugandi, C.A. Sullivan, Trees, forests and water: Cool insights for a hot world, Global Environmental Change, 43, 2017, pp 51-61, <https://doi.org/10.1016/j.gloenvcha.2017.01.002>
8. Ornstein, L., Aleinov, I., Rind, D., Irrigated afforestation of the Sahara and Australian Outback to end global warming. Climatic Change (2009) 97: 409. <https://doi.org/10.1007/s10584-009-9626-y>
9. Bastin, Jean-Francois, Yelena Finegold, Claude Garcia, Danilo Mollicone, Marcelo Rezende, Devin Routh, Constantin M. Zohner, Thomas W. Crowther, The global tree restoration potential, Science, Vol. 365, Issue 6448, pp. 76-79, <https://DOI.org/10.1126/science.aax0848>
10. Ter Maat, H.W., Hutjes, R.W.A. , Ohba, R., Ueda, H., Bisselink, B., Bauer, T., Meteorological impact assessment of possible large scale irrigation in Southwest Saudi Arabia, Global and Planetary Change, Volume 54, Issues 1–2, 2006, Pages 183-201, <https://doi.org/10.1016/j.gloplacha.2006.01.018>.
11. Puma, M. J., and B. I. Cook (2010), Effects of irrigation on global climate during the 20th century, J. Geophys. Res., 115, D16120, doi:10.1029/2010JD014122.
12. Pei, L., N. Moore, S. Zhong, A.D. Kendall, Z. Gao, and D.W. Hyndman, 2016: Effects of Irrigation on Summer Precipitation over the United States. J. Climate, 29, 3541–3558
13. Kang, S., & Eltahir, E. A. B. (2019). Impact of irrigation on regional climate over Eastern China. Geophysical Research Letters, 46, 5499– 5505. https://doi.org/10.1029/2019GL082396, <https://doi.org/10.1175/JCLI-D-15-0337.1>
14. Sagiv, A., Christofides, P.D., Cohen, Y., Semiat, R., On the analysis of FO mass transfer resistances via CFD analysis and film theory, Journal of Membrane Science, Volume 495, 2015, Pages 198-205, <https://doi.org/10.1016/j.memsci.2015.08.022>
15. Yip, N.Y., Tiraferri, A., Phillip, W.A., Schiffman, J.D., Hoover, L.A., Kim, Y.C., Elimelech, M., Thin-Film Composite Pressure Retarded Osmosis Membranes for Sustainable Power Generation from Salinity Gradients, Environmental Science & Technology 2011 45 (10), 4360-4369, <https://doi.org/10.1021/es104325z>
16. Helfer, F., Lemckert, C., Anissimov, Y.C., Osmotic power with Pressure Retarded Osmosis: Theory, performance and trends – A review, Journal of Membrane Science, 2014, 453, 337-358, <https://doi.org/10.1016/j.memsci.2013.10.053>.
17. Ganora, D., Dorati, C., Huld, T. A., Udias, A., & Pistocchi, A. (2019). An assessment of energy storage options for large-scale PV-RO desalination in the extended Mediterranean region. Scientific Reports, 9(1), 16234. <https://doi.org/10.1038/s41598-019-52582-y>
18. Voutchkov, N. Desalination engineering: planning and design. (McGraw Hill Professional, 2012).
19. Pistocchi, A., Dorati, C., Huld, T. and Salas Herrero, M., Hydro-economic assessment of the potential of PV-RO desalinated seawater supply in the Mediterranean region: Modelling concept and analysis of water transport costs, EUR 28982 EN, Publications Office of the European Union, Luxembourg, 2018, ISBN 978-92-79-77211-5, doi:10.2760/8455, JRC109866.

1. European Commission, Joint Research Centre (*) Corresponding author [↑](#footnote-ref-1)
2. Federal University of Parana', Brazil [↑](#footnote-ref-2)
3. LUT University, Finland [↑](#footnote-ref-3)
4. Politecnico di Torino, Italy [↑](#footnote-ref-4)
5. CEAM, Spain [↑](#footnote-ref-5)
6. Seawater Greenhouse, UK [↑](#footnote-ref-6)
7. Water Development Department, Cyprus [↑](#footnote-ref-7)
8. Water and Energy Agency, Malta [↑](#footnote-ref-8)
9. Technion, Israel [↑](#footnote-ref-9)
10. SWPC, United Arab Emirates [↑](#footnote-ref-10)
11. RWTH Aachen, Germany [↑](#footnote-ref-11)
12. CIEMAT, Spain [↑](#footnote-ref-12)
13. <https://web.stanford.edu/group/Urchin/mineral.html> [↑](#footnote-ref-13)
14. Wedepohl H (1995) The composition of the continental crust. Geochimica et Cosmochimica Acta 59: 1217–1239. For Au: Krauskopf, K.B., Bird, D.K., Introduction to geochemistry, 3rd edition. McGraw - Hill, New York, 1995. [↑](#footnote-ref-14)
15. Source: USGS Mineral commodities summary 2019, <https://www.usgs.gov/centers/nmic/mineral-commodity-summaries> , unless otherwise indicated. [↑](#footnote-ref-15)
16. Indicative – no data available [↑](#footnote-ref-16)
17. Share of production from Goodenough et al., 2017 <https://doi.org/10.1007/s11053-017-9336-5>, using rare earth element production data from <https://web.mit.edu/12.000/www/m2016/finalwebsite/problems/ree.html>. [↑](#footnote-ref-17)
